# Revenge Fantasies After Experiencing Traumatic Events: Sex Differences

**DOI:** 10.3389/fpsyg.2019.00886

**Published:** 2019-05-09

**Authors:** Limor Goldner, Rachel Lev-Wiesel, Guy Simon

**Affiliations:** The Emili Sagol Creative Arts Therapies Research Center, University of Haifa, Haifa, Israel

**Keywords:** desire for revenge, fantasies of revenge, sex differences, trauma history, gender, sex

## Abstract

Experiences of humiliation, unjust hurt caused by another or anger naturally, elicit the desire to seek revenge and fantasies of revenge. The current study examined the associations between a history of traumatic events and feelings of injustice and levels of desire for revenge-seeking and fantasies of revenge. Specifically, it tested whether feelings of injustice mediated the associations between the number of past traumatic events and the desire for revenge or revenge fantasies. Based on recent studies showing that retaliatory violence is gendered, sex differences in levels of feelings of injustice, desire for revenge, and the presence of revenge fantasies were explored, as well whether participants’ sex conditioned the mediation models. The results showed positive associations between feelings of injustice and the desire for revenge and revenge fantasies. The mediation model indicated that feelings of injustice mediated the associations between the number of previous traumatic events and the desire for revenge or revenge fantasies. Men had higher levels of revenge fantasies than women, whereas women tended to perceive revenge as pointless. A sex effect was found for the mediation model, which revealed significant regressed models for women but not for men. The clinical implications are discussed.

## Introduction

Revenge is defined as “an action in response to some perceived harm or wrongdoing by another party that is determined to inflict damage, injury, discomfort, or punishment to the party judged responsible” (Aquino et al., 2001, pp. 53). Revenge is considered to have a biological, evolutionary, and instinctive basis in that its roots stem from people’s basic animal fighting instincts (Haen and Weber, 2009; Fatfouta and Merkl, 2014). Modern Western thought considers revenge taboo and encourages forgiveness instead (Grobbink et al., 2015), though emotionally and even politically it is still a potent force in society (Fischer et al., 2010). Moreover, researchers have suggested that forgiveness may be conditioned by culture, religiosity, and spiritual beliefs (Sandage et al., 2003; Schultz et al., 2010) but the findings are mixed (e.g., Suwartono et al., 2007; Paz et al., 2008).

Several goals are fulfilled by the revenge (Orth, 2004). Revenge is intended to re-equilibrate the gains and losses caused by an assault. When the individual perceives him or herself as severely harmed by another person, who is held responsible for this harm to the individual’s ego, the ego assesses the harm as morally wrong and unjustified, and therefore develops a motivation for retaliation (Orth, 2004; McClelland, 2010). This comes from the belief that the perpetrator should not profit from his or her wrongdoings; many people believe that the perpetrator deserves to be subjected to equal harm to that caused by him or her. The desire for revenge is also intended to re-equilibrate power and allow the victim to regain a sense of control over his or her circumstances (Salter, 2014). This is particularly important, since power and control, the perception of one’s ability to influence others, are a basic human need (Salter, 2014; Lillie and Strelan, 2016).

## The Desire for Revenge

Experiences of humiliation, unjust hurt by another or anger naturally elicit the desire to seek revenge (Haen and Weber, 2009; Morrissette, 2012; Fatfouta and Merkl, 2014). This desire is considered to be a universal personal response in all human cultures (McClelland, 2010). The desire for revenge; namely, to cause the perpetrator to suffer (Watson, 2015), does not cease until it is recognized and released in one way or another (Fatfouta and Merkl, 2014). The greater the harm and transgression caused to the victim, and the more the victim perceives the perpetrator’s responsibility for the harm, the greater the desire for revenge (van Denderen et al., 2014). The desire to seek revenge first appears during childhood (Haen and Weber, 2009). Children as young as nine are capable of retaliatory decision-making. They can consider factors such as whether the harm was purposeful, the type of retaliation desired, and the age difference between the perpetrator and the victim (Haen and Weber, 2009; Lillie and Strelan, 2016). However, the urge to retaliate for wrongs persists throughout adulthood (Bloom, 2001).

Regardless of whether the victim has an emotional connection to the perpetrator, the level of the desire for revenge depends on the severity of the transgression toward the victim (Schumann and Ross, 2010; Morrissette, 2012; McCullough et al., 2013). The level of the desire for revenge also impacts the behavior of the victim toward or in the presence of the perpetrator. This can range from attempts at total avoidance to an obsession with the perpetrator and the hurt (Grobbink et al., 2015; Watson, 2015). However, when the victim knows the perpetrator and has an emotional attachment, the level of revenge is thought to be contingent upon the desire to preserve the relationship with the perpetrator and feelings toward him or her. The greater the desire to preserve the relationship, the greater the tendency to either deny the hurt or forgive the perpetrator (Watson et al., 2016).

## Revenge Fantasies

Whereas most people in the Western world do not actually engage in vengeance, many tend to fantasize after being treated unjustly. Revenge fantasies are defined as actual descriptive thoughts on how to get even with the perpetrator (Horowitz, 2007; Haen and Weber, 2009). Revenge fantasies often serve to calm the negative feelings of frustration, humiliation, and insult by virtually punishing the perpetrator and settling the score between the victim’s suffering and the perpetrator’ actions (Haen and Weber, 2009). These fantasies may provide the victim with a form of sadistic pleasure and enable closure (Haen and Weber, 2009). Victims who suffer from feelings of powerlessness and humiliation sometimes comfort themselves with the pseudo-power of revenge fantasies (Knoll, 2010; Seebauer et al., 2014; Lillie and Strelan, 2016). Revenge fantasies exist at all ages: children use revenge fantasies to disavow their inability to act (and mourn) and disguise their feelings of shame (Haen and Weber, 2009).

## The Moderating Role of Sex

Gender stereotypes serve as powerful factors in organizing individuals’ identity. These stereotypes are preconceived ideas whereby men and women arbitrarily assigned characteristics and roles determined and limited by their sex (Heilman, 2001; Eagly and Karau, 2002). They prescribe and proscribe how men and women should and should not behave (Prentice and Carranza, 2002). Western norms for men endorse emotional stoicism, competition, agency, status striving, and toughness (Kiselica et al., 2008; Kiselica and Englar-Carlson, 2010) whereas women should be caring, communal, and modest, but not controlling, aggressive dominant or stubborn (Moss-Racusin et al., 2010).

In this context, recent research shows that retaliatory violence including revenge is gendered (Mullins et al., 2004) with women adopting a submissive position and tend to avoid aggression and retaliation while men are encouraged to exhibit aggressive behaviors and revenge (Feld and Felson, 2008). For example, women with higher affective dysregulated temperaments are more likely to exhibit revenge fantasies. However, these fantasies are directed against the self in the form of suicidal ideation, planning, or attempts (Serafini et al., 2012). Women’s self-harm can be reflected in the phenomenon of self-silencing, which refers to the intentional withholding of thoughts and feelings from relationship partners as a way of nourishing those relationships and circumventing possible loss, rejection, and conflict (Jack, 1991; Jack and Dill, 1992). These thoughts and feelings, which are manifested in various types of behaviors including the denial of expression of emotions or beliefs, presenting a submissive exterior to the public despite feeling hostility and anger, putting one’s needs ahead of the self-needs are mostly characterized by women and reflect women’ strategies to cope with patriarchal systems that limit women’s choices (Jack and Ali, 2010; Brody et al., 2014).

In contrast, since manhood is widely viewed as an elusive status (it must be earned) and tenuous (it must be demonstrated repeatedly through actions), men’s aggressive behaviors, including revenge, can be interpreted as men’s attempts to prove their manhood while mitigating their concerns about their status, especially when it has been threatened (Vandello et al., 2008; Bosson et al., 2009; Bosson and Vandello, 2011).

Empirically, studies have shown that men are likely to be socially rewarded for aggressive, vengeful behavior in cultures where men have privileged access to high-status positions (Mullins et al., 2004; Wilkowski et al., 2012). Men tend to view violent retaliation as a vital tool for their survival (Hamama-Raz et al., 2008). Men who are victims of violent action or humiliation also use aggression to retaliate or deter further victimization (Mullins et al., 2004; Wilkowski et al., 2012). They are less able to forgive slights and offenses, which thus leads to more pronounced traumatic experiences and a greater desire for revenge than in young women (Mullins et al., 2004; Hamama-Raz et al., 2008).

## The Current Study

**Figure 1:**
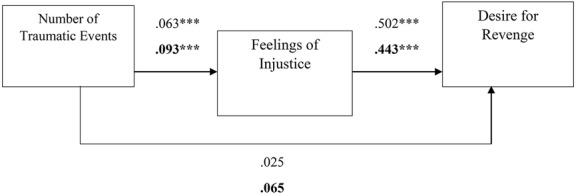
The estimations of the mediation model to predict desire for revenge. Note: ***p < 0.001, *N entire sample* = 278, *N = women sample* = 176, the black fonts represent women’s estimates.

**Figure 2:**
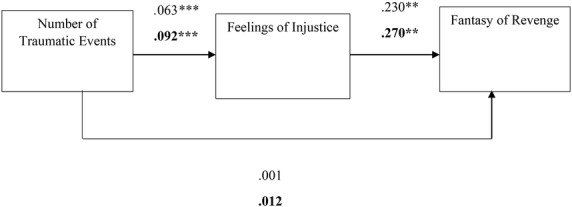
The estimations of the mediation model to predict fantasy of revenge. Note: ***p < 0.001, **p < 0.01, *N entire sample* = 278, *N = women sample* = 176, the black fonts represent women’s estimates.

**Figure 3:**
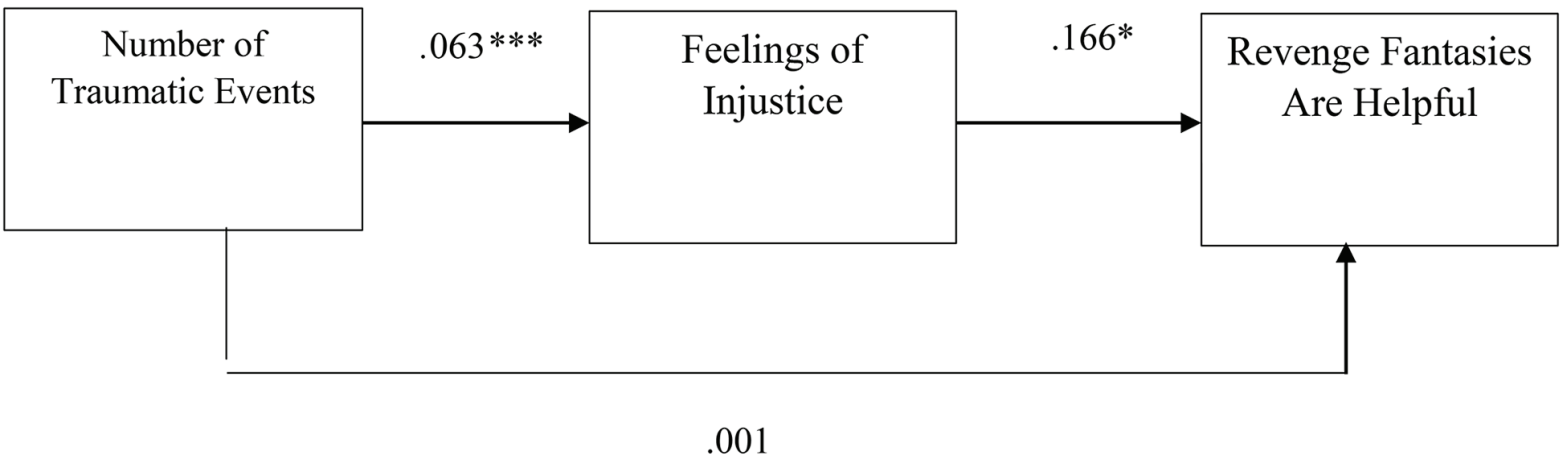
The estimations of the mediation model to predict the perception that revenge fantasies are helpful. Note: ***p < 0.001, *p < 0.05, *N entire sample* = 278.

**Figure 4:**
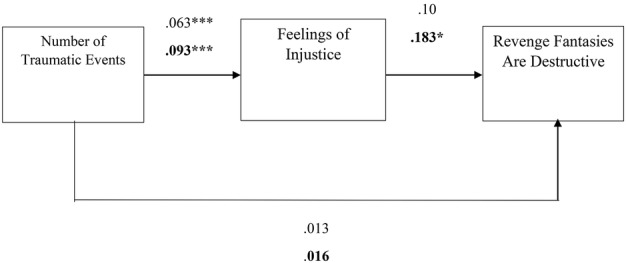
The estimations of the mediation model to predict the perception that revenge fantasies are destructive. Note: ***p < 0.001, *p < 0.05, *N entire sample* = 278, *N = women sample* = 176, the black fonts represent women’s.

Overall, the literature suggests that the desire for revenge and revenge fantasies serve as a form of narcissistic repair after experiences of harm and transgression while enabling acceptance and redefinition, encouraging progression, and reinforcing ego stability (McClelland, 2010; Seebauer et al., 2014; van Denderen et al., 2014; Watson et al., 2016). Nevertheless, to the best of our knowledge, the associations between a person’s history of traumatic events, feelings of injustice, and the desire for revenge and revenge fantasies have never been investigated. Thus, the first aim of the current study was to examine whether a history of trauma and feelings of injustice are associated with levels of desire for revenge and fantasies of revenge. In addition, differences between levels of feelings of injustice, the desire to seek revenge, and the presence of revenge fantasies were explored as a function of participants’ sex. Furthermore, an attempt was made to identify whether feelings of injustice served as an internal mechanism that elicits the desire for revenge and revenge fantasies using two mediation models (see Figures 1, 2). Given the distinction between the sexes with regard to revenge in the literature, it also examined whether men and women differ in their levels of desire for revenge and revenge fantasies. Finally, using two mediation models were used to examine whether participants’ sex would moderate the relationship between a history of traumatic events and feelings of injustice, which in turn would manifest in different levels of revenge fantasies or desires for revenge (see Figures 3, 4).

Three hypotheses were formulated: (1) Higher feelings of injustice and a more traumatic history should be correlated with higher levels of desire for revenge and more revenge fantasies. (2) The desire for revenge, destructive revenge fantasies, and the belief that revenge fantasies will help cope should differ according to sex. Specifically, it was hypothesized that women would have lower levels of desire for revenge and destructive fantasies than men, and lesser beliefs in the need for revenge and its contribution to the healing process than men. (3) Sex should moderate the associations between participants’ traumatic history and feelings of injustice, which thus should mediate the relationships between a history of traumatic events and the desire for revenge and revenge fantasies.

## Materials and Method

### Participants and Procedure

Two hundred seventy eight Hebrew-speaking adult men (*n* = 102) and women (*n* = 176) participated in the study. The mean age was 35.9 (*SD* = 10.9); 39.8% were married, and 81% had a college education. In terms of history of trauma, 43.4% reported of having been sexually abused, and 55.1% had experienced physical assault.After obtaining ethical approval from the ethics committee for research on human subjects of the Faculty of Welfare and Health Sciences of the University of Haifa, an anonymous online questionnaire (*via* Qualtrics) using validated questionnaire was administrated to the participants who were recruited through social media. All measures were independently translated into Hebrew from the English original by two translators, who are experts in the field of children at high risk and are native speakers of Hebrew. Then, their translations were compared, disagreements were discussed, and a final version was constructed. The presentation page of the questionnaire provided information about the goals of the study, a consent form, and a link to the online questionnaire. Participants were assured of the confidentiality of their responses. All the participants signed the online written consent form. There were no missing data. A power analysis using the Gpower computer program indicated that a total sample of 89 would be needed to detect large effects (*ƒ* ^2^ = 0.30) with 95% power using a linear multiple regression with an alpha of 0.05.

### Measures

#### The Traumatic Life Event Questionnaire

The Traumatic Life Event Questionnaire (TLEQ) is a 16-item self-report measure of 15 types of potentially traumatic events (e.g., natural disasters, motor vehicle accidents involving injuries or death, severe physical assault by an acquaintance or stranger, childhood physical abuse) and a final, open-ended event question that assesses exposure to some other life-threatening or profoundly disturbing event(s) (Kubany et al., 2000). For each item, participants are asked to indicate whether or not they have experienced any of these traumatic events. The inventory was shown to have adequate to excellent temporal stability (Kubany et al., 2000) and construct validity for the association between acknowledgment of exposure to more traumatic events on the TLEQ and increased likelihood of PTSD symptomatology (Kubany et al., 2000; Gray et al., 2004; Dedert et al., 2009).

#### The Injustice Experiences Questionnaire

The Injustice Experiences Questionnaire (IEQ) is a 12-item measure used to assess pain-related injustice perceptions (Sullivan et al., 2008). The IEQ measures cognitions associated with unfairness, perceived severity, and the irreparability of loss and blame (e.g., “It all seems so unfair”, “My life will never be the same”). Participants respond on a 5-point Likert-type scale ranging from 0 = “never” to 4 = “all the time.” The questionnaire was shown to have excellent internal and test-re-test reliabilities as well as construct validity for the association between the IEQ score and catastrophic thinking, fear of movement/re-injury, depression, and pain severity. The internal reliability (Cronbach’s alpha) in the current study was 0.90.

#### The Desire for Revenge (Transgression-Related Interpersonal Motivations, Inventory)

The revenge subscale from the transgression-related interpersonal motivations (TRIM) inventory was used (McCullough et al., 1998). It consists of five items assessing the respondents’ desire to seek revenge against someone, who committed a specific transgression (e.g., “I will make him or her pay.”). Participants are asked to rate their level of agreement with each item from strongly disagree (1) to strongly agree (5). This subscale has been shown to exhibit positive correlations with offense-specific rumination, negative affectivity and self-deception, and negative correlations with empathy and relational closeness. The internal reliabilities (Cronbach’s alpha) are reported to range from 0.85 to 0.93, with test-retest reliabilities in the range of 0.44–0.65 (McCullough et al., 1998). The internal reliability (Cronbach’s alpha) in the current study was 0.91.

#### The Revenge Fantasy Inventory for Adolescents

Twenty-two items from the revenge fantasy inventory for adolescents (RFI-J) were modified to assess attitudes toward revenge fantasies in adolescents (Warncke et al., 2015). The original questionnaire consisted of two sections (18 and 6 items rated on a 4-point Likert-type scale (1–“Does not apply” to 4–“fully applies”). The first section asked the participants about their feelings and thoughts about revenge fantasies for past injustice (e.g., “I refrain from revenge because it won’t change anything”). The second deals with imagined revenge fantasies (e.g., “I imagine harming this person physically”). Four factors were obtained: emotional regulation, rejection of revenge, negative emotionality, and readiness to act. Positive correlations have been reported between the RFI and measures of aggression and physical and psychological complaints.

In the present study, the items were modified for adults, translated into Hebrew by two native speakers and adapted to Israeli culture, for a total of 17 items from the original first part of the questionnaire and five items from the second. Two statements were removed: “my concepts of revenge fantasies amuse me,” “I imagine this person in an embarrassing situation” to strengthen and clarify the questionnaire.

As in the original research, a SPSS Principal Axis Factoring extraction method with VARIMAX rotation was used to identify different content areas around which the fantasies were directed (to what extent revenge helps, revenge as a necessary healing process, revenge is destructive). The factor loading matrix for this final outcome of three factors is presented in Table 1, which explained 49% of the variance. Composite scores were created for each of the three factors, based on the mean of the items, which had their primary loadings on each factor. The first factor was termed “Help” and was composed of seven items (2, 5, 9, 12, 14, 15, and 17) with factor loading ranging from 0.45 to 0.74, and an internal consistency (Cronbach’s alpha) of 0.90. A higher score indicated that thoughts of revenge helped in the healing process. The second factor was termed “Unnecessary” and consisted of six items (1, 3, 6, 8, 13, and 16) with factor loading ranging from 0.45 to 0.74 and an internal consistency (Cronbach’s alpha) of 0.79. Higher scores indicated a belief that revenge is unnecessary. The last factor was termed “Destructive” and consisted of four items (4, 7, 10, and 11) with factor loadings ranging from 0.50 to 0.80, and an internal consistency (Cronbach’s alpha) of 0.72. Higher scores indicated a belief that revenge thoughts are destructive. The factor analysis was also validated by performing a SEM confirmatory factor analysis (CFA) using the AMOS statistical package. Fit indices appeared to show a good fit to the data [*χ*^2^ (14, 153) =1.84, *p* < 0.01, CFI = 0.95, TLI = 0.94, RMSEA = 0.06, SRMS = 0.06]. Dimensions were calculated by computing the mean of the items that comprised the factor. A mean score was calculated for the second part of the questionnaire. A higher score indicated that vengeful thoughts helped the respondent cope with trauma. The internal reliability of the second part of the questionnaire in the current study was 0.90.

**Table 1:** Factor loading for 17 items, extracting three factors.

	Revenge thoughts are helpful (Help)	Revenge is unnecessary (Unnecessary)	Revenge thoughts are destructive (Destructive)
Item_1	−0.04	**0.45**	0.16
Item_2	**0.70**	−0.21	0.00
Item_3	−0.09	**0.74**	0.14
Item_4	−0.14	0.19	**0.69**
Item_5	**0.80**	−0.10	0.00
Item_6	−0.01	**0.69**	0.15
Item_7	0.04	0.23	**0.80**
Item_8	−0.17	**0.71**	0.30
Item_9	**0.69**	0.12	−0.03
Item_10	0.01	0.18	**−0.79**
Item_11	−0.11	0.27	**0.50**
Item_12	**0.79**	−0.06	−0.07
Item_13	−0.16	**0.73**	0.12
Item_14	**0.87**	−0.12	−0.07
Item_15	**0.84**	−0.04	−0.10
Item_16	−0.04	**0.69**	0.16
Item_17	**0.80**	−0.23	−0.07

The values in bold indicate the factor loading more than 0.40.

## Results

To examine the first hypothesis, relationships between the number of traumatic events, feelings of injustice (IEQ), the desire for revenge (TRIM), and fantasies of revenge, a series of Pearson product-moment correlation coefficients was calculated. As can be seen in Table 2, and in line with the first hypothesis, positive low correlations were found between levels of feelings of injustice (IEQ) and the desire for revenge (TRIM) (*r* = 0.30, *p* < 0.001), fantasies of revenge (FAN) (*r* = 0.21, *p* < 0.001) and the perception that revenge helps in the healing process (HELP) (*r* = 0.13, *p* < 0.05). Contrary to expectations, no correlations were found between the number of experienced traumatic events and the desire for revenge or fantasies of revenge. However, the number of experienced traumatic events (TLEQ) was correlated with feelings of injustice (IEQ). *t*-tests were conducted to examine differences in levels of desire for revenge and revenge fantasies between participants with and without a history of sexual or physical abuse.

**Table 2:** Descriptive statistics and Pearson correlation coefficients.

Variable	TLEQ	IEQ	TRIM	FAN	HELP	UNNEC	DEST
TLEQ							
IEQ	0.23***						
TRIM	0.12	0.30***					
FAN	0.08	0.21***	0.40***				
HELP	−0.04	0.13^*^	0.51***	0.52***			
UNNECESSARY	0.10	0.02	−0.35***	−0.27***	−0.24***		
DESTRUCTIVE	0.07	0.11	−0.14*	−0.05	−0.15*	0.51^**^	
*M*	4.39	2.00	2.56	2.20	2.09	2.69	2.27
*SD*	2.52	0.76	1.23	0.86	0.78	0.69	0.67

Note: *N* = 278. TLQ, Traumatic Life Event Questionnaire; IEQ, Injustice Experience Questionnaire; TRIM, desire to revenge; FAN, second part; HELP, revenge thoughts help; UNNECESSARY, revenge is unnecessary; DEST, revenge thoughts are destructive thoughts.

* *p* < 0.05,

** *p* < 0.01,

*** *p* < 0.001.

### Sex Differences

Sex was significantly correlated with history of sexual abuse and physical assault with female participants more sexually [*n* = 89, Chi-square (1) = 5.23, *p* < 0.05] and physically abused [*n* = 100, Chi-square (1) = 32.20, *p* = <0.001] than the male participants.

In addition, to examine the differences in revenge scores (TRIM, desire for revenge; FAN, fantasy inventory; HELP, revenge thoughts help; UNNECESSARY, revenge is unnecessary; DESTRUCTIVE, revenge thoughts are destructive) between sexes *t-test* analyses were conducted. Comparative women’s and men’s scores are presented in Table 3. As predicted, significant differences between women and men were found in levels of revenge fantasies (FAN) and in the perception that revenge is unnecessary (UNNECESSARY). Men had higher levels of revenge fantasies than women and women perceived revenge as pointless. Contrary to expectations, no significant differences were found between men and women for levels of desire for revenge (TRIM), the perception of revenge fantasies as helpful with coping with the trauma (HELP), or the perception of revenge fantasies as destructive (DESTRUCTIVE). In addition, women reported higher levels of feelings of injustice (IEQ) than men.

**Table 3:** Sex effect in variables study.

	*M* (*SD*)		*t*	*df*	*η*^2^	Mean differences	95% CI	
	Women *n* = 176	Men *n* = 102					Lower bound	Upper bound
TLEQ	4.43 2.51	4.30 2.55	−0.44	208.21	0.00	−0.14	−0.76	0.48
IEQ	2.11 0.71	1.82 0.66	3.53**	223.07	0.04	−0.30	−0.47	−0.13
TRIM	2.51 1.20	2.63 1.29	−0.73	198.36	0.00	0.11	−0.19	0.42
FAN	2.34 0.87	2.12 0.76	2.10*	190.06	0.02	0.22	0.01	0.42
HELP	2.03 0.77	2.21 0.79	1.84	205.82	0.01	0.18	−0.01	0.37
UNNECESSARY	2.96 0.69	2.80 0.69	−2.17*	209.48	0.02	−0.19	−0.37	−0.02
DESTRUCTIVE	2.28 0.72	2.25 0.74	−0.36	205.81	0.00	−0.03	−0.22	0.15

Note: *N* = 278. TLQ, Traumatic Life Event Questionnaire; IEQ, Injustice Experience Questionnaire; TRIM, desire to revenge; FAN, second part; HELP, revenge thoughts help; UNNECESSARY, revenge is unnecessary; DEST, revenge thoughts are destructive thoughts.

* *p* < 0.05,

** *p* < 0.001.

### Mediation Models

To test the relationship between the participants’ history of traumatic events and the desire for revenge, level of fantasies of revenge, or the various types of fantasies for revenge *via* feelings of injustice, five mediation models were examined using a bootstrapping procedure with 5000 bootstrap samples employing the SPSS Macro for multiple regressions as stipulated in Preacher et al. (2007) mediation model (model 4). The bootstrap procedure is considered superior to other methods such as the Sobel test. The model assumes that the relationship between the independent variable and the mediator is contingent on the level of the moderator, with no need for preconditioned direct links between the dependent and the independent variables or between the independent variable and the mediator or the moderator (Hayes, 2009).

The bootstrapping analyses revealed three significant mediation effects for desire for revenge (TRIM) (*estimate* = 0.032, *SE* = 0.011, *p* = 0.000, 95% CI 0.013, 0.058), fantasies of revenge (FAN) (*estimate* = 0.014, *SE* = 0.006, *p* = 0.000, 95% CI 0.006, 0.029) and the perception that revenge fantasies are helpful (HELP) (*estimate* = 0.011, *SE* = 0.006, *p* = 0.000, 95% CI 0.002, 0.026). Specifically, a large number of previous traumatic events contributed to the development of feelings of injustice (*estimate* = 0.063, *p* = 0.000), which subsequently contributed to high levels of desire for revenge (*estimate* = 0.502, *p* = 0.000), fantasies of revenge (*estimate* = 0.230, *p* = 0.001) and the perception that revenge fantasies are helpful (*estimate* = 0.166, *p* = 0.016) (for the estimates of the models see Figures 1, 3). The direct effects were not found to be significant.

To test for the putative moderation of participants’ sex in the mediation models, we compared the measurement models between women and men separately. The data revealed significant indirect effects for women in predicting the desire for revenge (*estimate* = 0.041, *SE* = 0.016, *p* = 0.000, CI 0.015, 0.077) and fantasies of revenge (*estimate* = 0.025, *SE* = 0.008, *p* = 0.000, CI 0.011, 0.047) *via* feelings of injustice in women. The indirect effect for men was not found to be significant.

Specifically, a large number of previous traumatic events was positively correlated with feelings of injustice (*estimate* = 0.093, *p* = 0.000), which in turn was positively correlated with the desire for revenge (*estimate* = 0.443, *p* = 0.000) and fantasies of revenge (*estimate* = 0.270, *p* = 0.000) (see Figures 1, 2). Moreover, the bootstrapping analyses revealed a significant mediation effect for the desire for revenge (*estimate* = 0.041, *SE* = 0.016, *p* = 0.000, CI 0.015, 0.077) in women. A large number of previous traumatic events was positively correlated with feelings of injustice (*estimate* = 0.093, *p* = 0.000), which in turn was positively correlated with the notion that revenge fantasies are destructive (*estimate* = 0.183, *p* = 0.024). Again, the direct link was not significant (see Figure 4).

## Discussion

The results of the present study revealed positive associations between feelings of injustice and the desire for revenge, and between the latter and revenge fantasies. However, there was no direct link between the number of experienced traumatic events and the desire for revenge and revenge fantasies. Nevertheless, the mediation models showed that when participants had feelings of injustice they tended to indicate a desire for revenge, fantasies of revenge, and the perception that revenge fantasies were helpful in their healing process, which may encourage them to remedy the injustice caused to them. Thus, the level of feelings of injustice rather than the number of traumatic events may function as an internal mechanism for the development of the desire for revenge and its accompanying fantasies.

The fact that perceived feelings of injustice were positively correlated with the desire for revenge and fantasies of revenge is consistent with previous studies (Orth, 2004; Haen and Weber, 2009; McClelland, 2010; Morrissette, 2012; Fatfouta and Merkl, 2014; van Denderen et al., 2014), suggesting that the desire for revenge is a universal, cross-cultural response in humans (McClelland, 2010). Thus, the desire for revenge and fantasies of revenge may play a central role in counteracting negative emotions such as frustration and humiliation experienced by the victim (Horowitz, 2007). Through this desire and fantasies, individuals can imagine that the perpetrator has been punished and that justice has been rendered in the sense that the victim has attacked the perpetrator (Haen and Weber, 2009). In this way, the desire for revenge can serve as a means of rehabilitation and self-healing by instilling a sense of power and control through the planning of vengeance, even if there is no actual act of revenge (Morrissette, 2012; Berger, 2014). Future studies should explore this supposition further.

Similar to the findings reported in Mullins et al. (2004) and Wilkowski et al. (2012), who found sex differences in revenge fantasies and vengeful acts, in the current study women expressed greater feelings of injustice than men, but men tended to have greater revenge fantasies than women. In addition, women perceived revenge as pointless even though they reported more experiences of sexual abuse and higher levels of feelings of injustice. However, the mediation model showed that when taking feelings of injustice into account, women permitted themselves to develop a desire for revenge and fantasies of revenge. However, women still perceived these fantasies of revenge as dangerous in that they also evoked feelings of guilt and shame. As mentioned, a significant aspect of men’s identity is preserving sense of manhood and men’s status by actively using violent behaviors (Bosson and Vandello, 2011). In addition, certain cultures such as Mediterranean cultures are characterized by the notion of male honor. In these cultures, men are demanded to be hypersensitive to threats that damage their reputation, and are expected to restore their honor through the use of violence. By contrast, women are supposed to be submissive, modest, and avoidant (Vandello and Cohen, 2003). Thus, consistent with these claims, the findings here suggest a moderating role of gender identification as explaining men’s desire for revenge. While men are entitled to exhibit overt hostility and retaliation as result of an abusive environment, women perceived these acts as dangerous and meaningless.

### Study Limitations and Future Studies

Several limitations of the current study should be acknowledged. First, the convenience sample limits its generalizability. In addition, since the questionnaire was administered and filled in *via* social media, there was no control over the identity of the participants, their sincerity, or their motivations. The current study used a cross-sectional design, and the analyses as well as the structural equation model were based on correlational data, which could not chart the development of feelings of injustice or the desire for revenge after traumatic events. Furthermore, this study used a binary definition of sex, which may have missed some of the subtler differences between genders and sexual orientations known today. As far as we know, there is no literature on differences in revenge fantasies in LGBT individuals. The same can be said for race and socio-economic status. Studying these differences may further the effectiveness of therapy in these specific groups.

As noted above, previous studies such as Wilkowski et al. (2012) and Hamama-Raz et al. (2008) have shown that the desire for revenge is culture-dependent. The current study examined specific differences between people in terms of revenge fantasies and mechanisms of coping with traumatic victimization. While the current study focused on sex differences, cross-cultural studies on this topic might yield interesting results with practical, culturally-sensitive implications. The same methods as used in this study could be adapted for this type of research. This is particularly important as the literature on these differences is sparse and possibly outdated. Another future direction would be to examine the intersection between sex and culture in terms of coping mechanisms after traumatic victimization, especially for revenge fantasies. Since therapy needs to be sensitive not only to sex but also to race, class, and culture, this type of research is crucial to furthering the effectiveness of therapeutic interventions in these cases.

### Practical Implications

Despite these limitations, several implications can be drawn from the findings. First, the study points to the significance of feelings of injustice as an internal mechanism for evoking a desire for revenge and associated fantasies, which may endow the victim with a sense of control after the trauma. Although the natural tendency to seek revenge and revenge fantasies tend to be regarded in the literature as a negative component while the concept of forgiveness is seen as a positive step towards healing (Hargrave and Anderson, 2013; Tener and Eisikovits, 2017) along with the conversion of anger and resentment toward the perpetrator or the self into positive feelings such as compassion and empathy (McCullough et al., 2013), when practitioners develop intervention programs for individuals, who have experienced various traumatic events they may benefit by viewing the role of feelings of injustice, the desire for revenge and fantasies of revenge as necessary ingredients for healing prior to forgiveness.

The finding suggests that men tend to have higher levels of desire for revenge and revenge fantasies should be noted when working with male victims, to aim toward a better alternative to releasing these feelings of injustice. To improve wellbeing in women, especially those who have experienced traumatic events, intervention programs should be developed to help resolve the dissonance between feelings of injustice and fantasies of revenge and the feeling that these are pointless and destructive. Thus, the results of our study suggest that there is no “one size fits all” theory of revenge desire and fantasy and highlight the need for researchers to investigate further sex differences with the aim of developing a gender-sensitive therapy.

## Ethics Statement

The research reported here was conducted in accordance with American Psychological Association’s ethical standards.

## Author Contributions

GS gathered the data. All the authors analyzed the data and were involved in writing the article.

### Conflict of Interest Statement

The authors declare that the research was conducted in the absence of any commercial or financial relationships that could be construed as a potential conflict of interest.
